# Regioisomeric distribution of 9‐ and 13‐hydroperoxy linoleic acid in vegetable oils during storage and heating

**DOI:** 10.1002/jsfa.8766

**Published:** 2017-11-27

**Authors:** Marc Pignitter, Mathias Zaunschirm, Judith Lach, Laura Unterberger, Antonio Kopic, Claudia Keßler, Julia Kienesberger, Monika Pischetsrieder, Manfred Eggersdorfer, Christoph Riegger, Veronika Somoza

**Affiliations:** ^1^ Department of Physiological Chemistry, Faculty of Chemistry University of Vienna Vienna Austria; ^2^ Department of Chemistry and Pharmacy University of Erlangen‐Nürnberg Erlangen Germany; ^3^ Department of Human Nutrition and Health DSM Nutritional Products Ltd Kaiseraugst Switzerland

**Keywords:** lipid hydroperoxides, linoleic acid, heating, storage, vegetable oil

## Abstract

**BACKGROUND:**

The oxidative deterioration of vegetable oils is commonly measured by the peroxide value, thereby not considering the contribution of individual lipid hydroperoxide isomers, which might have different bioactive effects. Thus, the formation of 9‐ and 13‐hydroperoxy octadecadienoic acid (9‐HpODE and 13‐ HpODE), was quantified after short‐term heating and conditions representative of long‐term domestic storage in samples of linoleic acid, canola, sunflower and soybean oil, by means of stable isotope dilution analysis–liquid chromatography‐mass spectroscopy.

**RESULTS:**

Although heating of pure linoleic acid at 180 °C for 30 min led to an almost complete loss of 9‐HpODE and 13‐HpODE, heating of canola, sunflower and soybean oil resulted in the formation of 5.74 ± 3.32, 2.00 ± 1.09, 16.0 ± 2.44 mmol L^–1^ 13‐HpODE and 13.8 ± 8.21, 10.0 ± 6.74 and 45.2 ± 6.23 mmol L^–1^ 9‐HpODE. An almost equimolar distribution of the 9‐ and 13‐HpODE was obtained during household‐representative storage conditions after 56 days, whereas, under heating conditions, an approximately 2.4‐, 2.8‐ and 5.0‐fold (P ≤ 0.001) higher concentration of 9‐HpODE than 13‐HpODE was detected in canola, soybean and sunflower oil, respectively.

**CONCLUSION:**

A temperature‐dependent distribution of HpODE regioisomers could be shown in vegetable oils, suggesting their application as markers of lipid oxidation in oils used for short‐term heating. © 2017 The Authors. *Journal of The Science of Food and Agriculture* published by John Wiley & Sons Ltd on behalf of Society of Chemical Industry.

## INTRODUCTION

After purchase, the consumer usually stores edible oils at home for several months. This storage condition induces lipid oxidation in oils.^1^ Lipid oxidation leads to the formation of a broad range of different molecules. The structural heterogeneity of the products requires the application of several methods to study the progress of lipid deterioration.^2^ One of the most commonly studied indicators of the oxidative status of oils is the peroxide value.^3^ The peroxide value comprises the sum of lipid hydroperoxides formed upon lipid oxidation, although it does not provide any structural information in detail.

Health effects of lipid hydroperoxides very likely depend on their structure. Studies claimed that dietary hydroperoxides and advanced lipid oxidation end products are associated with the progression of diabetes mellitus^4^ and atherosclerosis.^5^, ^6^ Although the dietary intake of oxidized fat was associated with detrimental health effects,^7^ it has also been reported that dietary administration of hydroxy‐ or hydroperoxy octadecadienoic acid might cause beneficial effects, such as the reduction of cholesterol and triglyceride levels.^8^ Hydroperoxy octadecadienoic acid was demonstrated to activate the peroxisome proliferator activated receptor‐*α* by binding to peroxisome proliferator‐activated receptor‐*α* protein, which forms a complex with retinoid X receptor.^8^ This complex binds to specific DNA sequences and thereby stimulates the transcription of genes involved in fatty acid catabolism.

The health risks associated with a high dietary intake of lipid oxidation products are still controversially discussed, especially because there are no estimates for dietary intakes of individual lipid hydroperoxides. For example, linoleic acid, which represents one of the major unsaturated fatty acids in many edible oils, can be oxidized to several different linoleic acid hydroperoxides, depending on the type of chemical oxidation.^3^ The different mechanisms of chemical oxidation, autoxidation and photosensitized oxidation, lead to a different product spectrum of linoleic acid hydroperoxides.^9^ Excellent recent reviews of the mechanisms of lipid oxidation are available in the literature.^10^, ^11^ Briefly, the major regioisomers of autoxidized linoleic acid comprise 9‐ and 13‐hydroperoxy linoleic acid (9‐HpODE and 13‐HpODE). In addition to autoxidation, linoleate can also be subjected to singlet oxygen‐mediated photooxidation, leading to the generation of 9‐, 10‐, 12‐ and 13‐hydroperoxy linoleate.^3^ Furthermore, the geometric isomers, 10*E*, 12*Z* and 10*E*,12*E* for 9‐hydroperoxy linoleic acid, as well as 9*Z*,11*E* and 9*E*,11*E* for 13‐hydroperoxy linoleic acid, need to be considered.^3^ Besides chemical oxidation, linoleic acid can also be oxidized enzymatically.^12^, ^13^ The type of isomers of enzymatically oxidized linoleic acid mainly depends on the type of lipoxygenase. The formation of the regioisomeric linoleic acid hydroperoxides depends on pH, temperature and, most makedly, on reaction time.^12^ However, studies investigating the health effects of the individual linoleic acid hydroperoxides are scarce. Thus, it is of major relevance to differentiate between the linoleic acid hydroperoxide isomers formed during the oxidative deterioration of vegetable oils.

Lipid oxidation also occurs when edible oils are used by consumers for frying foods, although the product mix is much more complex. As with autoxidation, high‐temperature treatment of vegetable oils leads not only to the formation of lipid hydroperoxides, but also to the more rapid decomposition to secondary products such as alcohols and aldehydes. Thermal scissions of acyl chains generate radicals that recombine to form polymers. Hydrolytic cleavage in the presence of water releases free fatty acids, which are also subjected to lipid oxidation and saponification.^14^ As a result of high temperatures, dehydration causes further decomposition of peroxides to yield polymers, epoxides, alcohols and hydrocarbons.

Although the detection of lipid peroxides is considered to be a valuable measure for tracking oil deterioration during storage, it is not recommended for evaluating the oxidative quality of frying oils because peroxides decompose at high temperature and with repetitive heating over time.^15^ However, the oxidative stability of oils after heating for a limited period of time, as performed by consumers at home, has not been investigated so far. We hypothesize that short‐term exposure of vegetable oils to high temperatures for up to 30 min, such as is applied under household frying conditions, might lead to detectable concentrations of the two regioisomers 9‐ and 13‐HpODE.

Because of the detrimental and beneficial health effects of oxidized lipids, the identification and quantitation of dietary intake of individual oxidized lipids is required. We aimed to identify the contribution of 9‐HpODE and 13‐HpODE to the total peroxides formed after household‐representative storage and heating conditions. We hypothesized that HpODEs are relevant markers for monitoring lipid oxidation during domestic storage, as well as under heating conditions.

## MATERIALS AND METHODS

### Materials, chemicals and study oils

Refined soybean, sunflower (high oleic) and canola oil were purchased from local supermarkets in Austria. Immediately after purchase, the oils were filled in transparent 0.5‐L polyethylene bottles, which were kindly provided by Radlberger AG (Unterradlberg, Austria) and the study was started on the same day. All vegetable oils were required to have a peroxide value <2 meq O_2_ kg^–1^ to be included in the study. The bottles were subjected to illumination by the fluorescent lamp Tornado from Philips (Eindhoven, The Netherlands) with 1450 lm and 103 W covering a spectrum from 400 to 650 nm. All chemicals were ordered from Sigma‐Aldrich (Vienna, Austria) or Carl Roth (Karlsruhe, Germany).

### Storage conditions for vegetable oils

A total of 460 ± 0.1 g of each vegetable oil (*n* = 4) was filled in transparent bottles and stored under household‐representative conditions at 22 ± 2 °C for 56 days. Specifically, the bottles were arranged to allow equal distance of 15 cm between the bottles and 34 cm between the bottles and the light source. The vegetable oils were exposed to cold fluorescent light for 12 hours daily, whereas, during the remaining 12 h, the oils were kept in the dark for 56 days. To mimic consumer handling, the bottles were opened once a week for sampling of 40 mL of oil, thereby increasing the headspace volume. On days 1, 7, 14, 28 and 56, the samples were stored at –80 °C until analyses. All vegetable oils were purchased and measured within their expiry dates and, at the end of the storage period (day 56), they still had 7.5 ± 0.5 months left to reach their expiry date.

### High temperature conditions for vegetable oils and free linoleic acid

For short‐term experiments, a total volume of 50 µL of linoleic acid was transferred to a 1.5‐mL brown glass vial, which was left open. The surface of the linoleic acid amounted to 0.63 cm^2^. Vegetable oils at a volume of 20 mL were placed into a dish with an inner diameter of 9 cm leading to a surface of 64 cm^2^. The linoleic acid and vegetable oils were heated in an oil bath at 180 °C in the dark for a maximum of 30 min prior to storage at –80 °C until analyses.

For long‐term experiments, linoleic acid (50 µL) was loaded into glass (180 °C) or 500‐µL Eppendorf (40 and 99 °C) vials, which were incubated at 40, 99 and 180 °C for 0, 4, 8, 16 and 24 h. Subsequently, the samples were stored at –80 °C until analyses.

### Determination of the total peroxides in vegetable oils

The total lipid peroxides were determined by measuring the peroxide value in accordance with the AOCS official method Cd 8‐53^16^ and as described in detail recently.^1^ Briefly, potassium iodide was added to the oil samples dissolved in a mixture of glacial acetic acid and chloroform (3:2, v/v). The extent of oxidation of iodide by the lipid peroxides was determined by titration with 0.1 N standardized sodium thiosulfate. The peroxide value was expressed as meq O_2_ kg^–1^.

### Synthesis of 13‐hydroperoxy‐(9Z,11E)‐octadecadienoic acid (13‐HpODE)

13‐HpODE was synthesized as described by Grosch^17^ and Pignitter *et al*.^18^ with slight modifications. Linoleic acid (200 mg) was added to 8 mL of 0.1% Tween‐80 solution. This mixture was dissolved in 2 mL of 1 N NaOH, diluted to 200 mL with 0.02 mol L^–1^ sodium borate buffer (pH 9.0), cooled in an ice bath and then bubbled with oxygen for 5 min. Soybean lipoxygenase (type I, 4.2 mg dissolved in 1 mL of 0.02 mol L^–1^ sodium borate buffer, pH 9.0) was added and the reaction mixture was incubated for 2 h at room temperature in the dark. The reaction was stopped by adding 2 N HCl to pH 3 and hydroperoxides were extracted with 3 × 200 mL of diethyl ether; the ether extracts were then combined, washed with 2 × 200 mL of double‐distilled water, and dried with sodium sulfate. Finally, the extract was transferred to a rotary evaporator for removal of solvent.

For determination of purity, the synthesis product was analyzed by LC–MS (LCMS‐2020; LabSolutions software; Shimadzu, Shimadzu, Vienna, Austria) coupled with a MS detector with an electrospray ionization (ESI) source. A methanol gradient (flow rate: 0.5 mL min^–1^) was used to separate the synthesis product on a C18 column (Luna C18 100 A, 250 × 3 mm, 5 µm; Phenomenex, Torrance, CA, USA), starting with methanol/double‐distilled water 50/50 (vol%), increasing to 100% methanol over 20 min, holding for 10 min. The starting conditions were set again until 40 min. Positive and negative total ion current [TIC(±)] and negative single ion monitoring [SIM(−)] were recorded with the MS settings: nebulizing gas flow, 1.5 L min^–1^, drying gas flow, 10 L min^–1^, desolvation line temperature, 250 °C, heating block temperature 250 °C, interface voltage 4.5 kV, scan range: *m*/*z* 50–700, SIM: *m*/*z* 311 (13‐HpODE), *m*/*z* 279 (linoleic acid). After determining the purity (95.1%) by analyzing the ^1^H‐nuclear magnatic resonance (NMR) spectra and the TIC chromatograms, structure identification was performed by 1D/2D NMR (Bruker Avance III; Bruker Rheinstetten, Germany) and spectra were analyzed using SpinWorks, version 3.1.8.1 (University of Manitoba, Winnipeg, MB, USA). ^1^H‐NMR (d_6_‐acetone, 500 MHz): *δ* (ppm) 4.39 [dt,1H, CH,C (13)], 5.50 [dt, 1 H, J = 10.9 Hz; CH, C (9)], 5.58 [dd, 1 H, J = 15.3 Hz; CH, C (12)], 6.02 [dd, 1 H, J = 10.9 Hz; CH, C (10)], 6.58 [dd, 1 H, J = 15.3 Hz; CH, C (11)].^19^, ^20^ The synthesis and identification of the isotope labeled standard (^13^C_18_)‐13‐hydroperoxy‐(9*Z*,11*E*)‐octadecadienoic acid ([^13^C_18_]‐13‐HpODE, purity: 92.0%) were carried out analogous to the synthesis of 13‐HpODE using ^13^C_18_‐linoleic acid (99 atom% ^13^C_18_) as the substrate.

### Synthesis of 9‐hydroperoxy‐(10E,12Z)‐octadecadienoic acid (9‐HpODE)

9‐HpODE was synthesized according to Grosch^17^ and Spiteller^13^ using the procedure described above for 13‐HpODE, except that a 1% Tween‐80 solution, 0.1 mol L^–1^ potassium phosphate buffer (pH 6.3) and 1 N KOH for adjusting the pH value were used. For the incubation, potato lipoxygenase (388 µL, 5 kU) was used. The synthesis product was purified once using a semipreparative HPLC (Dionex Ultimate 3000; Thermo Fisher Scientific, Vienna, Austria) equipped with a C18 column (Luna C18 100 A; 250 × 3 mm, 5 µm; Phenomenex) and ultraviolet detector set at 234 nm. Products were eluted with a methanol gradient (flow rate 0.5 mL min^–1^): starting with methanol/double‐distilled water 50/50 (vol%), increasing to 100% methanol over 20 min, holding for 10 min. This was followed by a return to starting conditions over 35 min and holding for 5 min. The synthesis product eluted at 23 min and the corresponding fraction was collected. Synthesis product purity (95.0%) and structure were determined as described for 13‐HpODE. ^1^H‐NMR (d_6_‐acetone, 500 MHz): *δ* (ppm) 4.33 [dt,1H, CH, C (9)], 5.48 [dt, 1 H, J = 10.7 Hz; CH, C (13)], 5.63 [dd, 1 H, J = 15.3 Hz; CH, C (10)], 6.01 [dd, 1 H, J = 10.7 Hz; CH, C (12)], 6.58 [dd, 1 H, J = 15.3 Hz; CH, C (11)].^21^

### Oil sample preparation

The analysis of the primary peroxidation products of linoleic acid, 13‐HpODE and 9‐HpODE in the different stored oil samples was adapted from Salimon *et al*.^22^ and Browne and Armstrong.^23^ In a brown glass vial (20 mL), 28.05 µg of [^13^C_18_]‐13‐HpODE was added as an internal standard to 37 mg (39.8 µL for sunflower and soybean oil and 40.2 µL for canola oil) of oil sample and dissolved in 1 mL of ethanolic potassium hydroxide solution (2 mol L^–1^). To prevent oxidation during hydrolysis, the mixture was purged with nitrogen and sealed. Then, hydrolysis was performed in the dark at 60 °C in a water bath for 1.5 h. Afterwards, 1 mL of double‐distilled water was added and unsaponifiable components were extracted with 2 × 1 mL of hexane. Then, 120 µL of glacial acetic acid was added, extraction with 2 × 2 mL of hexane was performed and all of the hexane extracts were collected. After evaporating hexane with nitrogen, the residue was dissolved in 500 µL of ethanol and filtered through a nylon filter (0.2 µm).

### Quantitation of 9‐ and 13‐hydroperoxy octadecadienoic acid

The sample was analyzed using LC–MS (LCMS‐8040; Shimadzu) coupled with a tandem MS detector with an ESI source. An isocratic methanol gradient (60% methanol/40% double‐distilled water) with a flow rate of 0.5 mL min^–1^ was used (Kinetex F5, 100 A, 150 × 2.1 mm, 2.6 µm; Phenomenex). Multiple reaction mode [MRM(–)] was recorded with the MS settings: nebulizing gas flow, 3 L min^–1^; drying gas flow, 10 L min^–1^; desolvation line temperature, 250 °C; heat block temperature, 150 °C; CID gas, argon; collision energy, 15 V; MRM (precursor ion *m/z* > product ion *m/z*), 13‐HpODE (311 > 113), 9‐HpODE (311 > 185), [^13^C_18_]‐13‐HpODE (329 > 120).^24^ Quantitation of 13‐HpODE and 9‐HpODE was performed in MRM(–) mode using a stable isotope dilution assay selecting the fragment ions *m/z* 113 for 13‐HpODE (*y* = 0.4719*x* + 0.1929, *r* ^2^ = 0.99) *m/z* 185 for 9‐HpODE (*y* = 1.8819*x* + 0.9542, *r* ^2^ = 0.97) and *m/z* 120 for ^13^C_18_‐13‐HpODE, as internal standard. 13‐HpODE and 9‐HpODE in the sample were identified by comparing their retention times and fragmentation patterns with their corresponding synthesized standards. Furthermore, the MRM fragments *m/z* 113 and *m/z* 185, each likely formed by dehydration and scission of the double bond next to the oxygenated center in accordance with the suggested mechanism of a single or two proton shift, respectively,^25^ were chosen because of their selective formation within the corresponding linoleic acid hydroperoxide.^24^ Limit of detection (LOD) was determined by a signal‐to‐noise ratio of 3, whereas the limit of quantitation (LOQ) was calculated based on a signal‐to‐noise ratio of 10. The intra‐day and inter‐day repeatability of the method were determined for the oil samples spiked with 28.05 µg [^13^C_18_]‐13‐HpODE and showed a relative standard deviation of 8.47% and 9.63%, respectively (*n* = 21).

### Statistical analysis

All experiments were performed with three independent and two technical replicates. The results are expressed as the mean ± SD. Statistically significant differences were calculated using Sigma Plot, version 11.0 (Systat Software Inc., Chicago, IL, USA) applying one‐way analysis of variance (ANOVA) followed by Student–Newman–Keuls or Holm–Sidak post‐hoc tests. *P* < 0.05 was considered statistically significant.

## RESULTS

### Quantitation of regioisomers of linoleic acid hydroperoxides in vegetable oils stored under household conditions

**Figure 1:**
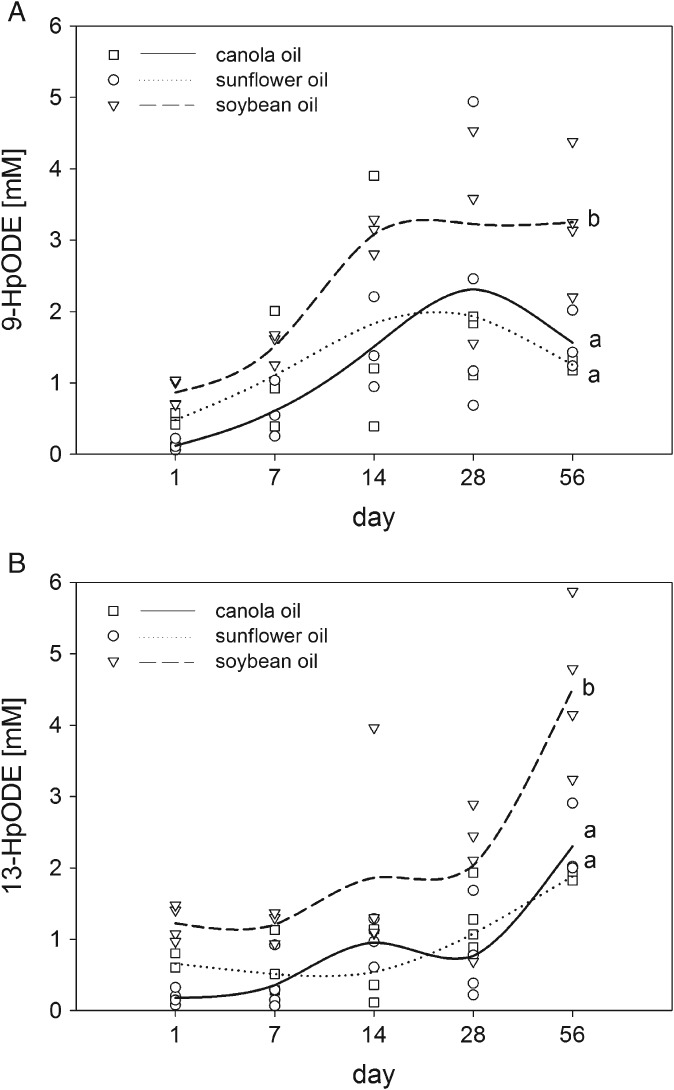
Quantitation of 9‐hydroperoxy octadecadienoic acid (9‐HpODE) (A) and 13‐HpODE (B) in canola, sunflower and soybean oil stored under household conditions for 56 days. Data are expressed as symbols and area under the curve (AUC) (n = 3 or 4). Statistically significant differences between the different oil types after 56 days were calculated by applying one‐way ANOVA following a Holm–Sidak post‐hoc test (P < 0.05) and are indicated by different lowercase letters (a,b).

For identification of the peroxidation products formed during storage in vegetable oils, the main primary products of lipid autoxidation of the most abundant polyunsaturated fatty acid, linoleic acid, 9‐HpODE and 13‐HpODE^3^, were quantified. Canola, sunflower and soybean oil were chosen as the study oils as a result of their high consumption and their different linoleic acid content.^26^ In canola, sunflower and soybean oil, the regioisomers, 9‐HpODE and 13‐HpODE, were quantified on days 1, 7, 14, 28 and 56 of storage under household‐representative conditions. It was demonstrated that the regioisomers increased after 56 days of domestic storage of the oils, yielding 1.25 ± 0.09, 1.56 ± 0.41 and 3.25 ± 0.89 mmol L^–1^ 9‐HpODE and 1.88 ± 0.06, 2.30 ± 0.53 and 4.50 ± 1.10 mmol L^–1^ 13‐HpODE in sunflower, canola and soybean oil, respectively (Fig. 1). The generation of 13‐HpODE did not significantly differ from the formation of 9‐HpODE in all oils stored under domestic conditions for 56 days (*P* > 0.05). On days 7, 14 and 28 of storage, there were no significant changes in the amount of 9‐HpODE and 13‐HpODE between the different oil types detected (*P* > 0.05). After 56 days of storage, the formation of 9‐HpODE and 13‐HpODE was higher in soybean oil compared to sunflower and canola oil (*P* < 0.05) because these regioisomeric hydroperoxides are predominantly formed during autoxidation of linoleic acid.^3^ The initial content of linoleic acid was the highest in soybean oil (55% of total fatty acids) compared to the sunflower (15% of total fatty acids) and canola oil (20% of total fatty acids).

**Figure 2:**
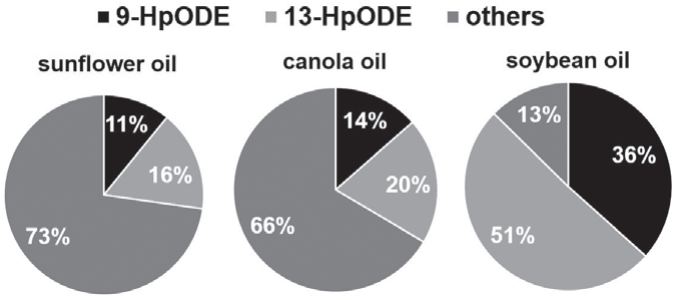
Percentage of 9‐hydroperoxy octadecadienoic acid (9‐HpODE) and 13‐HpODE on total peroxides formed in sunflower, canola and soybean oil stored under household conditions for 56 days. Data are expressed as the mean (n = 3).

Figure 2 demonstrates the proportion of 9‐ and 13‐HpODE to the total peroxides formed during domestic storage of the oils for 56 days. The contribution of 9‐ and 13‐HpODE to total peroxides in sunflower, canola and soybean oil amounts to 27%, 34% and 87%, respectively. The other hydroperoxides formed were determined by subtracting the 9‐ and 13‐HpODE from the total peroxide value. Depending on the oil type, up to 87% of lipid peroxides are 9‐HpODE and 13‐HpODE.

### Quantitation of 9‐ and 13‐HpODE in free linoleic acid subjected to thermal treatment

**Figure 3:**
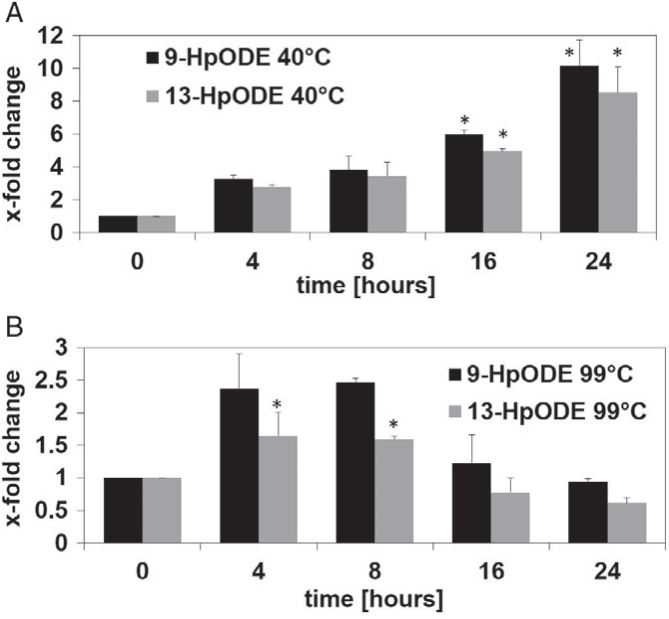
Quantitation of 9‐hydroperoxy octadecadienoic acid (9‐HpODE) and 13‐HpODE after thermal treatment of linoleic acid at 40 °C (A) and 99 °C (B) for 0, 4, 8, 16 and 24 h. Data are displayed as the fold change compared to a non‐treated control and are expressed as the mean ± SD (n = 3). Statistically significant differences to linoleic acid, which was not thermally treated, were calculated by applying one‐way ANOVA following a Student–Newman–Keuls post‐hoc test. Differences (P < 0.05) are indicated by asterisks (*).

**Figure 4:**
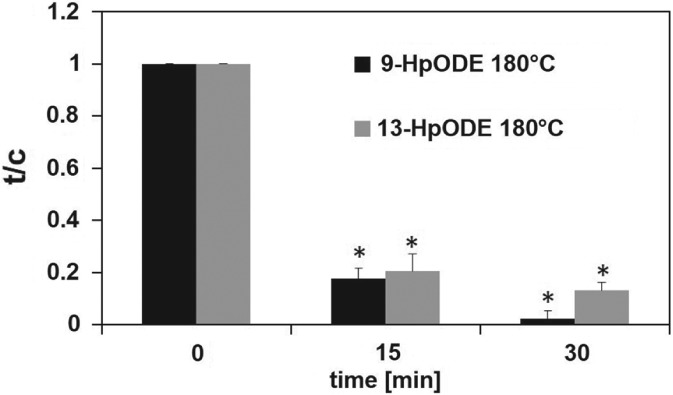
Quantitation of 9‐hydroperoxy octadecadienoic acid (9‐HpODE) and 13‐HpODE after thermal treatment of linoleic acid at 180 °C for 0, 15 and 30 min. Data are displayed as treated over control (t/c) and expressed as the mean ± SD (n = 3). Statistically significant differences to linoleic acid, which was not thermally treated (control), were calculated by applying one‐way ANOVA following a Student–Newman–Keuls post‐hoc test. Differences between treatment times versus no treatment (P < 0.05) are indicated by asterisks (*).

To investigate whether the regioisomers of linoleic acid hydroperoxides can also be used as reliable markers for the detection of lipid oxidation in frying oil, the formation of 9‐ and 13‐HpODE under different thermal conditions was first evaluated in free linoleic acid. The effect of thermal treatment of 50 µL of linoleic acid at 40, 99 and 180 °C on the formation of 9‐HpODE and 13‐HpODE was investigated. The temperatures were selected to mimic conditions during heating at low, moderate and high temperatures. At 40 °C, a 10‐ and eight‐fold increase of 9‐HpODE and 13‐HpODE, respectively, compared to non‐treated linoleic acid was observed after 24 h (Fig. 3A). At 99 °C, a 1.5‐fold rise of 13‐HpODE was obtained after 8 h, which declined below the initial concentration after 24 h (Fig. 3B). Incubation of linoleic acid at 180 °C for 4–24 h resulted in a complete loss of the regioisomers of the lipid hydroperoxides (data not shown) although the LOD was in the low nanomolar range, reaching 35 ± 9.1 nmol L^–1^ and 5.1 ± 0.7 nmol L^–1^ for 9‐ and 13‐HpODE, respectively. The LOQ was calculated to be 117 ± 31 nmol L^–1^ and 17 ± 2.3 nmol L^–1^ for 9 and 13‐HpODE, respectively. To investigate whether short‐term incubation of linoleic acid at 180 °C might lead to detectable concentrations of 9‐ and 13‐HpODE, the time of incubation was limited to a couple of minutes. Thermal treatment of linoleic acid at 180 °C for only 15 or 30 min also led to an almost complete loss of 9‐HpODE and 13‐HpODE (Fig. 4).

### Quantitation of 9‐ and 13‐HpODE in vegetable oils incubated at 180 °C

**Figure 5:**
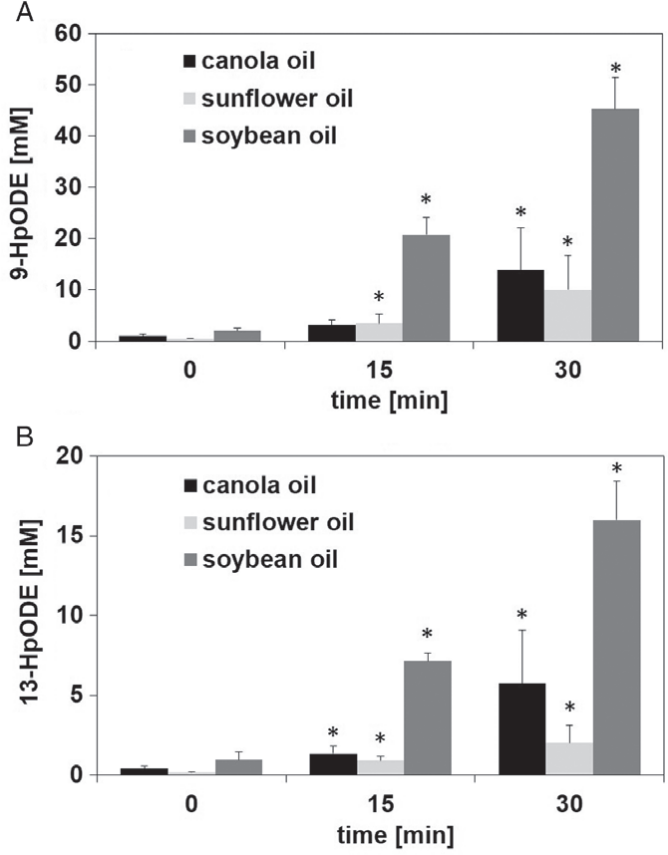
Quantitation of 9‐hydroperoxy octadecadienoic acid (9‐HpODE) (A) and 13‐HpODE (B) in canola, sunflower and soybean oil after heat treatment at 180 °C for 0, 15 and 30 min. Data are expressed as the mean ± SD (n = 3). Statistically significant differences to the respective vegetable oil without heat treatment were calculated by applying one‐way ANOVA following a Student–Newman–Keuls post‐hoc test. Differences between treatment times versus no treatment (P < 0.05) are indicated by asterisks (*).

To evaluate whether 9‐ and 13‐HpODE can be used as markers of lipid oxidation in oils utilized for domestic frying, we quantified the hydroperoxide isomers in vegetable oils after heat treatment at 180 °C for 0, 15 and 30 min (Fig. 5). By contrast to the experiments with the free linoleic acid, treatment of sunflower and soybean oil at 180 °C resulted in a significant rise of the 9‐ and 13‐HpODE already after 15 min. Thermal treatment of vegetable oils at 180 °C for 30 min induced an increase of 9‐HpODE to 13.8 ± 8.21, 10.0 ± 6.74 and 45.2 ± 6.23 nmol L^–1^ in canola, sunflower and soybean oil, respectively (Fig. 5A). Similarly, the concentration of 13‐HpODE increased after heat treatment of the oils at 180 °C for 30 min, albeit to a lesser extent, yielding approximately only 5.74 ± 3.32, 2.00 ± 1.09 and 16.0 ± 2.44 nmol L^–1^ in canola, sunflower and soybean oil, respectively (Fig. 5B).

## DISCUSSION

### Formation of 9‐ and 13‐HpODE in oils stored under domestic conditions

The oxidative stability of vegetable oils is determined by environmental factors, most importantly oxygen availability, temperature and light.^9^, ^10^ Although numerous studies have investigated the stability of vegetable oils, only a few studies have analyzed the effects of domestic storage and handling conditions on the oxidative status of the oil.^1^, ^27^–^30^ Recently, light was demonstrated to be the major elicitor of rancidity of edible oils stored under household conditions.^1^ Most of the studies published so far rely on the analysis of total peroxides, thereby providing no information about the specific lipid peroxides formed. The present study showed that 9‐ and 13‐HpODE increased almost equimolarly in vegetable oils during 56 days of storage under domestic conditions. It could also be demonstrated that the 9‐HpODE formed during the initial period of storage was partially degraded at the end of the storage period. It can be assumed that the generation and degradation of 9‐HpODE occur simultaneously during storage, albeit to a different extent, favoring degradation over the generation at the end of the 2‐month storage. According to the molecular mechanism of lipid oxidation, linoleic acid is oxidized to 9‐ and 13‐HpODE at a ratio of 1:1.^3^ Thus, the product distribution of oxidized linoleic acid is not substantially influenced by the oil matrix. It could also be demonstrated that the amount of 9‐ and 13‐HpODE formed in oil during storage was dependent on the fatty acid composition of the oil. Although storage of soybean oil rich in linoleic acid led to the highest concentration of the regioisomers, storage of canola oil and sunflower oil with the lowest concentration of linoleic acid showed the lowest concentration of these isomers. The relative amount of linoleic acid of all unsaturated fatty acids reached 16.7%, 22.5% and 64.6% in sunflower, canola and soybean oil. It can be expected that the proportion of linoleic acid to unsaturated fatty acids might be similar to the proportion of 9‐ and 13‐HpODE to non‐linoleic acid derived hydroperoxides. As a result, a similar albeit higher proportion of 9‐ and 13‐HpODE was obtained, confirming a preferred oxidation of linoleic acid compared to oleic acid. It is well known that bis‐allylic hydrogen atoms have lower C‐H bond energies than allylic hydrogen atoms, explaining the preferred abstraction of hydrogen from linoleic acid and a higher proportion of oxidized linoleic acid products.^31^

### 9‐ and 13‐HpODE as markers for oils exposed to short‐term heating conditions

Although, for evaluating the storage‐induced increase of lipid oxidation in edible oils, total peroxides, as well as the regioisomers, 9‐ and 13‐HpODE, can be considered as a valuable marker, they are not regarded as useful indicators for investigating the oxidative quality of frying oils subjected to thermal treatments.^14^ Thermal oxidation follows accelerated autoxidation.^14^ Although lipid hydroperoxides are formed in oils under thermal conditions, their degradation is enhanced at elevated temperatures. Thus, the characterization of frying oils is chiefly performed by analyzing the extent of polymerization and other secondary reaction products.^32^, ^33^ Frying conditions are not standardized, differing between large‐scale catering, industrial and domestic frying. Although, for large‐scale catering, the frying oil is usually subjected to high temperature for several hours, industrial frying uses the same oil only for a maximum of 5 h.^34^ In the case of domestic frying, the oil is treated for no longer than 30 min at elevated temperatures. Considering the different frying conditions, it might be conceivable that lipid hydroperoxides are relevant markers for examining the oxidative status of frying oil as well. In addition, uncovering the exposure to individual lipid hydroperoxides is required to investigate their health effects. The literature is scarce with regard to the influence of short‐term heating on the oxidative stability of oils. The present study showed that, at 40 °C, concentrations of 9‐ and 13‐HpODE increased significantly over a 24‐h incubation of free linoleic acid, whereas, at 180 °C, the degradation of the regioisomers of the hydroperoxides appears to be higher than the rate of its generation. Heat has been reported to induce the decomposition of lipid hydroperoxides to alkoxy and hydroxyl radicals.^9^ Therefore, evaluating the effect of long‐term treatment of vegetable oils at elevated temperatures cannot be achieved by analyzing lipid hydroperoxides. Even short‐term treatment of linoleic acid at 180 °C for up to 30 min led to an almost complete loss of 9‐ and 13‐HpODE in the present study. Interestingly, short‐term exposure of vegetable oils to 180 °C for up to 30 min resulted in an increased formation of 9‐ and 13‐HpODE. Thus, these regioisomers might be used as markers to study the progress of lipid oxidation in oils subjected to short‐term heating conditions. It is suggested that, in contrast to free linoleic acid, the rate of formation of 9‐ and 13‐HpODE is most likely higher than the rate of degradation in vegetable oils under household‐representative heating conditions. This discrepancy between the stability of the free linoleic acid and the stability of the edible oils processed for 30 min at 180 °C might be explained by the presence of minor compounds in the oil matrix, such as tocopherols, and the difference in the surface‐to‐volume ratio of the oil and the free linoleic acid.^35^–^37^ α‐Tocopherol was shown to have strong electron‐donating abilities, thereby converting lipid peroxyl radicals to lipid hydroperoxides by oxidation of tocopherol to α‐tocopheryl quinone.^36^ Besides the lipid hydroperoxide forming effect of tocopherols, lipids with a small sample size and a high surface‐to‐volume ratio were shown to react more efficiently with oxygen.^35^, ^37^ The surface‐to‐volume ratio of the linoleic acid sample was 12.6 cm^2^ mL^–1^, whereas for the vegetable oils it amounted 3.2 cm^2^ mL^–1^, which could also contribute to the observed difference in the stability between the free linoleic acid and vegetable oils under heating conditions. Thus, the oxidation of vegetable oils might be more limited compared to free linoleic acid. It might also be conceivable that oil triglycerides are hydrolyzed prior to propagation of autoxidation mechanisms under heating conditions,^14^ thereby slowing down the formation of linoleic acid hydroperoxides.

### Temperature‐dependent distribution of HpODE regioisomers

**Figure 6:**
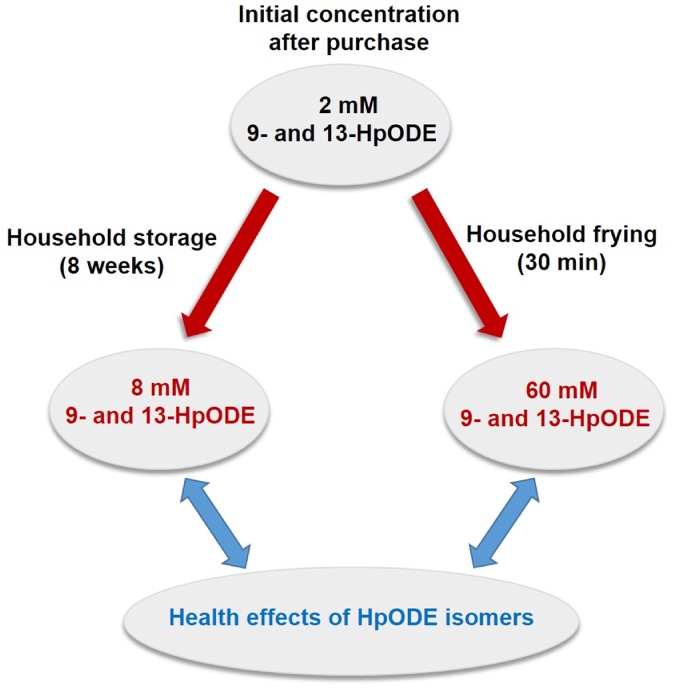
Quantitative data on the potential dietary intake of 9‐hydroperoxy octadecadienoic acid (9‐HpODE) and 13‐hydroperoxy octadecadienoic acid (13‐HpODE) concentrations from soybean oil.

The present study also revealed that the ratio of 9‐ and 13‐HpODE regioisomers detected appears to be dependent on the temperature, as is evident by comparing experiments at room temperature with frying temperature. In soybean oil, which is one of the most consumed vegetable oil worldwide, an initial concentration of approximately 2 mmol L^–1^ 9‐ and 13‐HpODE was detected (Fig. 6). After storage of the oil for 8 weeks under household conditions, the concentration of 9‐ and 13 HpODE increased to 8 mmol L^–1^. After heating of the oil under household conditions, a total of 60 mmol L^–1^ of these regioisomers could be measured in soybean oil. After 30 min at 180 °C, an approximately 2.8‐fold (*P* ≤ 0.001) higher concentration of 9‐HpODE compared to 13‐HpODE was obtained in soybean oil, whereas, at room temperature, almost equal amounts of 9‐ and 13‐HpODE were detected. Similar results were obtained for heated canola and sunflower oil at 180 °C for 30 min, reaching an approximately 2.4‐ and 5.0‐fold higher concentration 9‐HpODE than 13‐HpODE, whereas no significant difference was detected in the respective oils stored at room temperature. So far, it has been assumed that linoleic acid autoxidized equally to its main degradation products, 9‐ and 13‐HpODE.^3^ In the present study, we report a temperature‐dependent distribution of HpODE regioisomers in vegetable oils. The generation of 9‐HpODE or/and the decomposition of 13‐HpODE appears to be favored at elevated temperatures.

Quantitative analyses of both 9‐ and 13‐HpODE in heated vegetable oils allow the determination of the dietary load of these regioisomers by the consumption of french fries. A portion size of 150 g of french fries contains about 22 g of fat, which largely derives from the frying fat. Thus, by consumption of soybean oil, which has been subjected to domestic heating conditions for 30 min, a dietary intake of approximately 60 mmol L^–1^ 9‐ and 13‐HpODE, which corresponds to approximately 1.3 mmol (406 mg) for a typical serving of about 22 g of oil, is achieved.^38^, ^39^ Similar amounts of HpODEs and total peroxides were shown to exert proinflammatory effects.^40^ Next step will be to investigate the health effects of the HpODE isomers and its metabolites.

## CONCLUSIONS

Household storage‐induced oxidation of soybean oil led to the formation of mainly 9‐ and 13‐HpODE, which amounted to 87% of total peroxides. Typical household heating conditions resulted in an almost complete loss of 9‐ and 13‐HpODE when free linoleic acid was evaluated, whereas a significant increase of 9‐ and 13‐HpODE was demonstrated in commercial vegetable oils. Additionally, it could be shown that the distribution of the regioisomers, 9‐ and 13‐HpODE, does not differ under household storage conditions, whereas a different distribution could be detected during heating. 9‐ and 13‐HpODE are suggested as markers of lipid oxidation not only in oils subjected to domestic storage, but also in those used for short‐term heating.
